# Effect of ghrelin administration on postoperative inflammatory response and bodyweight loss in patients with oesophageal cancer undergoing oesophagectomy: a systematic review and meta-analysis

**DOI:** 10.1007/s00423-023-02970-w

**Published:** 2023-06-14

**Authors:** Elizabeth Forshaw, Shahin Hajibandeh, Shahab Hajibandeh

**Affiliations:** 1 School of Medicine, Cardiff University, Cardiff, UK; 2 Department of General Surgery, Royal Stoke University Hospital, Stoke-on-Trent, UK; 3 Department of General Surgery, University Hospital of Wales, Cardiff & Vale NHS Trust, Cardiff, UK

**Keywords:** Ghrelin, Oesophageal cancer, Oesophagectomy

## Abstract

**Objectives:**

To investigate the effect of postoperative ghrelin therapy on postoperative inflammatory response and bodyweight loss in patients undergoing an oesophagectomy for oesophageal cancer.

**Methods:**

We conducted a systematic search using electronic information databases in accordance to PRISMA standards to identify studies comparing outcomes after oesophagectomy in patients who were and were not administered ghrelin in the postoperative period. Meta-analysis of the outcomes using random effects modelling was conducted. The Cochrane collaboration’s tool and ROBINS-I tool were used for risk of bias assessment of the included studies.

**Results:**

Five studies including 192 patients were selected for analysis. Ghrelin therapy was associated with a significantly shorter duration of systemic inflammatory response syndrome (SIRS) (MD: − 2.72, *P* = 0.0001), lower CRP level on postoperative day 3 (MD: − 3.64, *P* < 0.0001), and less total bodyweight loss (MD: − 1.87, *P* = 0.14). There was no differences between the two groups in IL-6 level on postoperative day 3 (MD: − 19.65, *P* = 0.32), total lean body weight loss (MD: − 1.87, *P* = 0.14), total body fat loss (MD: 0.15, *P* = 0.84), pulmonary complications (OR: 0.47, *P* = 0.12), anastomotic leak (OR: 1.17, *P* = 0.78), wound complications (OR: 1.64, *P* = 0.63), postoperative bleeding (OR: 0.32, *P* = 0.33), arrhythmia (OR: 1.22, *P* = 0.77).

**Conclusions:**

Administration of ghrelin following oesophagoectomy may reduce duration of postoperative SIRS and bodyweight loss. Whether shorter duration of SIRS and less bodyweight loss resulted from postoperative ghrelin therapy can translate into improved morbidity or mortality outcomes remains unknown. There is a need for randomised controlled trials with robust statistical power to investigate the role of postoperative ghrelin therapy on morbidity and mortality outcomes in patients undergoing oesophagectomy.

## Introduction

In the absence of contraindications to surgery, oesophagectomy remains the mainstay curative treatment of oesophageal cancer [1]. However, oesophagectomy is one of the most invasive gastrointestinal surgeries and is associated with substantial postoperative morbidity and mortality. Notably, oesophagectomy commonly causes excessive systematic inflammatory response syndrome (SIRS) due to the increase in production of inflammatory markers such as cytokines TNF-alpha and IL-6 in the acute postoperative period [2]. These cytokines are thought to cause various postoperative complications in the acute phase, such as bodyweight loss, lung injury, and multi-organ failure [3]. Many studies have identified that these postoperative complications have a negative influence on patient quality of life [4] and contribute to poor prognosis following surgical resection [5].

Ghrelin is a peptide hormone produced predominantly by oxynitic glands in the gastric fundus of the stomach which has been identified as an endogenous ligand for growth hormone (GH) [6]. Ghrelin has several physiological functions, including the promotion of appetite signal in the hypothalamus and stimulation of gastrointestinal activity. Additionally, ghrelin is thought to have inhibitory effects on inflammatory cytokine production [7, 8]. Research has shown that patients who underwent oesophagectomy had decreased plasma ghrelin levels in the postoperative period [9]. The lower the level of ghrelin postoperatively was inversely correlated to an increased SIRS duration [10]. This observation warranted investigation into whether exogenous ghrelin administration may reduce excess cytokine production and shorten the duration of SIRS after oesophagectomy.

Several clinical studies have evaluated outcomes of postoperative administration of ghrelin in patients undergoing oesophagectomy. This would make performing a systematic review worthwhile for evidence synthesis. Therefore, in the present study, we aimed to perform a comprehensive review of the literature and conduct a meta-analysis of the outcomes of ghrelin administration in patients undergoing oesophagectomy.

## Methods

### Design and eligibility criteria

Selection of studies, data collection, outcome synthesis, and data analysis were done according to prespecified criteria which had been documented in a review protocol. This protocol was registered at the International Prospective Register of Systematic Reviews (registration number: CRD42022342474). The review conformed to the Preferred Reporting Items for Systematic Reviews and Meta-Analyses (PRISMA) statement standards [11].

Any comparative study (randomised controlled trials, prospective or retrospective cohort studies, and case-control studies) investigating the effects of ghrelin administration on post-operative outcomes were considered eligible as study design of interest. Participants of any age and gender who had undergone radical oesophagectomy and gastric tube reconstruction as curative treatment of oesophageal cancer were considered eligible as population of interest. Postoperative ghrelin administration of any dose, duration, or regimen was defined as intervention of interest; placebo or not receiving ghrelin therapy was defined as comparisons of interest. The primary outcome measure was postoperative inflammatory response [C-reactive protein (CRP) level on postoperative day 3, IL-6 level on postoperative day 3, and duration of SIRS]. The secondary outcome measures were total bodyweight loss, lean body weight loss, fat body weight loss, pulmonary complications, anastomotic leak, wound complications, bleeding, and arrhythmia.

### Search methods

A suitable and rigorous search strategy was developed by two independent authors using relevant search terms, keywords, thesaurus headings, and medical subject headings (MeSH) (Appendix I). The search was last applied on 18 June 2022 and no language constraints existed. The following sources were searched: the National Library of Medicine’s MEDLINE database using the PubMed Web-based search engine, the Cochrane Central Register of Controlled Trials (CENTRAL), Cumulative Index to Nursing and Allied Health Literature (CINAHL), Excerpta Medica database (EMBASE), The World Health Organization International Clinical Trials registry, European Association for Grey Literature Exploitation, System, International Standard Randomised Controlled Trial Number Registry, and ClinicalTrials.gov. Moreover, relevant articles were identified from reference lists of primary studies, systematic reviews, and meta-analyses relevant to our research topic.

### Selection of studies, data extraction, and assessment of risk of bias

The study selection step and subsequent data extraction step were undertaken by two independent reviewers (E.F. and S.H.). The above comprehensive search strategy was used to identify the titles and abstracts of the eligible literature for our study. Articles identified as suitable were then screened by reading the full texts, and if the study met our outlined eligibility criteria for the study, it was selected. An electronic data extraction spreadsheet was created, and the following data was extracted from each study: first author’s name, year, country of origin, journal of the published study, study design, sample size, description of included participants, ghrelin administration regimen, age, gender, tumour location, disease stage, field of lymph node dissection, neoadjuvant therapy, operative time, and blood loss. A third author acted as an adjudicator in the event of disagreements.

The Cochrane collaboration’s tool was applied to assess the risk of bias of the randomised trials by two independent authors; the tool has a role to verify quality of the study by ensuring there is random generation of group allocation (selection bias), ensuring that the trial is blind (performance bias), blinding the outcome of the assessment (detection bias), evaluating any incomplete outcome data (attrition bias), and ensuring there is no reporting bias such as only reporting selective outcomes. The bias of observational studies was assessed using the Risk Of Bias In Non-Randomized Studies of Interventions (ROBINS-I) tool [12]. This tool acts to evaluate whether bias is present in observational studies and how this affects the methodological quality of the study. It targets confounding, selection, classification, performance, attrition, detection, and outcome recall bias [13]. A separate and independent third author was used to act impartially in case of disagreements between the first two authors regarding bias.

### Summary measures, outcome synthesis, and sensitivity analyses

We used Review Manager 5.4.1 (RevMan, Version 5.4.1 Copenhagen, 2020) software to create a meta-analysis model to make comparisons between outcomes. Random effects modelling was used to determine odds ratio (OR) when assessing dichotomous outcomes and mean difference (MD) when assessing continuous outcomes. The ORs represented the odds of an adverse event happening during the postoperative period following oesophagectomy in participants who had been administered ghrelin therapy compared with those taking a placebo or receiving no ghrelin therapy during this period. An OR of < 1 meant that ghrelin treatment was favourable for this given outcome. The heterogeneity among studies for each of the outcomes was calculated and measured as *I*^2^ using Cochran *Q* test (*χ*2). We classified the heterogeneity of each study according to percentages with an *I*^2^ of between 0 and 25% being low heterogeneity, moderate was *I*^2^ from 25 to 75%, and when *I*^2^ was 75–100%, this meant there was a high heterogeneity. Publication bias was assessed visually by evaluating the symmetry of funnel plot for each outcome reported by at least 10 studies. Comparison meta-analysis model was based on 95% confidence level to demonstrate statistical significance.

Sensitivity analyses were planned and undertaken for outcomes reported by at least four studies. In order to identify whether any individual studies were disproportionately affecting the overall spread of the results, analysis was repeated for each outcome, excluding one contributing study each time and reviewing the spread of results and whether this changed. Moreover, we changed the summary measure from OR to risk ratio (RR) and risk difference (RD) to assess consistency of the findings. In addition, due to concern about potentially overlapping population between the studies of Yamashita (2) (2021) and Takata (2015), we repeated analyses after removing the study of Takata (2015). Removing the study of Takata (2015) did not affect the direction of the effect size for any of the outcomes. Finally, we undertook separate analyses for randomised controlled trials and studies at overall low risk of bias.

## Results

**Fig. 1:**
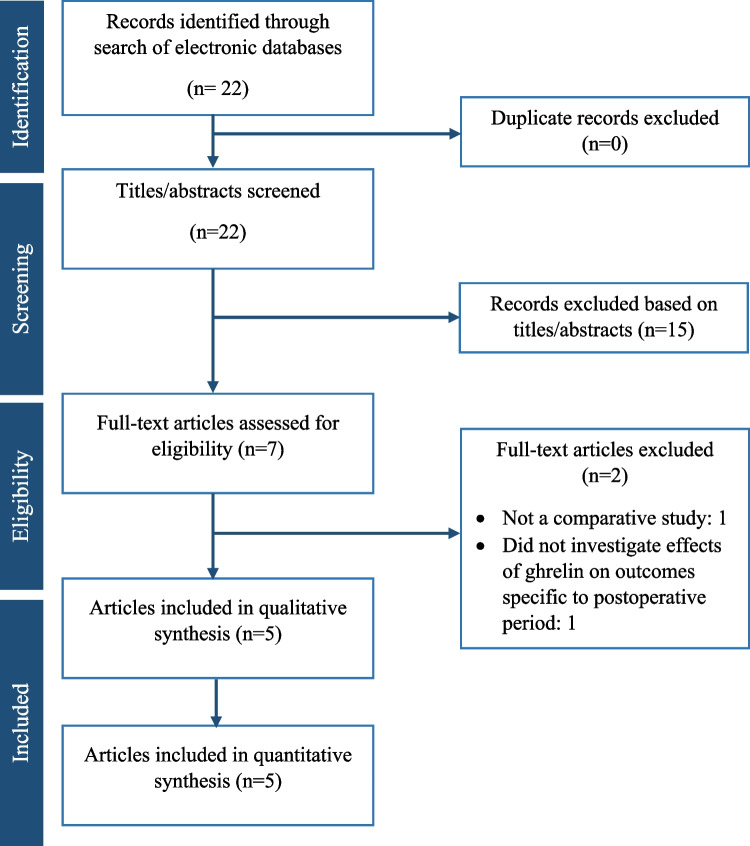
PRISMA flow chart

The search of electronic databases resulted in 22 articles from which we were able to immediately exclude 15 studies as they did not discuss a topic relevant to our study. The full text of the study was then read of the remaining seven articles, and following review, two more were excluded as one was not a comparative study and the other did not investigate the effect of ghrelin treatment specific to the postoperative period. Five articles remained [14–18] which met the eligibility criteria (Fig. 1). These included three randomised controlled trials [14, 16, 17] and two prospective cohort studies [15, 18] enrolling a total of 192 patients suitable for our meta-analysis (96 patients in the ghrelin group and the other 96 patients in the no ghrelin group). Information about each study including the design of the study, its publication date, the details of the study populations, and regimen of ghrelin therapy is presented in Table 1. The baseline demographics and clinical characteristics of the patients in each study, including age, gender, tumour location, disease stage, field of lymph node dissection, and use of neoadjuvant chemotherapy, are reported in Table 2.

**Table 1:** Baseline characteristics of the included studies

Study	Yamashita 2021	Yamashita (2) 2021	Takata 2015	Takata (2) 2015	Yamamoto 2009
Country	Japan	Japan	Japan	Japan	Japan
Journal	*Anticancer research*	*Esophagus*	*Surgery Today*	*Annals of Surgery*	*Surgery*
Study design	RCT	Prospective cohort	Prospective cohort	Phase II RCT	Phase II RCT
Recruitment period	February 2017–August 2018	May 2010–August 2011	May 2008–July 2009 May 2010–August 2011	April 2012–September 2013	October 2007–December 2008
Description of included population	Patients with thoracic oesophageal cancer undergoing radical oesophagectomy and gastric tube reconstruction	Patients with thoracic oesophageal cancer undergoing radical oesophagectomy and gastric tube reconstruction	Patients with thoracic oesophageal cancer undergoing radical oesophagectomy and gastric tube reconstruction	Patients with thoracic oesophageal cancer undergoing radical oesophagectomy and gastric tube reconstruction	Patients with thoracic oesophageal cancer undergoing radical oesophagectomy and gastric tube reconstruction
Sample size					
Total	52	40	40	40	20
Ghrelin therapy group	26	20	20	20	10
No ghrelin therapy group	26	20	20	20	10
Type of ghrelin regimen	0.5 μg/kg/h continuously for 7 days	3 μg/kg bd for 10 days or 0.5 μg/kg/h continuously for 5 days	3 μg/kg bd for 10 days or 0.5 μg/kg/h continuously for 5 days	0.5 μg/kg/h continuously for 5 days	3 μg/kg bd for 10 days

*RCT,* randomised controlled trial

**Table 2:** Baseline characteristics of the included population (ghrelin vs no ghrelin)

Study	Age^*^	Male gender	Operation	Neoadjuvant therapy	Tumour location			Disease stage				Field of lymph node dissection		Operative time (min)^*^	Intraoperative blood loss (mL)^*^
					Upper thorax	Middle thorax	Lower thorax	Stage I	Stage II	Stage III	Stage IV	2-Field	3-Field		
Yamashita 2021	68.0 (41–8) vs 68.5 (53–83)	22/26 vs 24/26	3-stage oesophagectomy with gastric tube reconstruction and cervical anastomosis	20/26 vs 17/20	NR	NR	NR	9/26 vs 9/26	3/26 vs 10/26	12/26 vs 5/26	2/26 vs 2/26	NR	NR	525.5 (371–828) vs 526 (338–728)	255 (30–1105) vs 255 (40–810)
Yamashita (2) 2021	62.5 (50–80) vs 66 (47–73)	17/20 vs 16/20	3-stage oesophagectomy with gastric tube reconstruction and cervical anastomosis	20/20 vs 18/20	9/20 vs 4/20	9/20 vs 9/20	2/20 vs 7/20	1/20 vs 1/20	4/20 vs 6/20	12/20 vs 8/20	3/20 vs 5/20	11/20 vs 10/20	9/20 vs 10/20	445 (359–596) vs 462.5 (353–574)	590 (250–1270) vs 605 (360–960)
Takata 2015	63.3 ± 8 vs 64.2 ± 7.4	17/20 vs 16/20	3-stage oesophagectomy with gastric tube reconstruction and cervical anastomosis	20/20 vs 18/20	2/20 vs 4/20	9/20 vs 9/20	9/20 vs 7/20	1/20 vs 1/20	4/20 vs 8/20	14/20 vs 10/20	1/20 vs 1/20	9/20 vs 10/20	11/20 vs 10/20	457.8 ± 60.6 vs 463.7 ± 53.8	593 ± 242 vs 635 ± 211.1
Takata (2) 2015	65.0 ± 6.5 vs 65.8 ± 6.0	19/20 vs 18/20	3-stage oesophagectomy with gastric tube reconstruction and cervical anastomosis	19/20 vs 18/20	3/20 vs 2/10	9/20 vs 11/20	8/20 vs 7/20	2/20 vs 3/20	7/20 vs 7/20	6/20 vs 7/20	5/20 vs 3/20	8/20 vs 9/20	12/20 vs 11/20	420.1 ± 40.5 vs 432.4 ± 59.1	463.5 ± 227.7 vs 483.8 ± 238.8
Yamamoto 2009	63 ± 6 vs 65 ± 6	9/10 vs 9/10	3-stage oesophagectomy with gastric tube reconstruction and cervical anastomosis	7/10 vs 9/10	2/10 vs 1/10	4/10 vs 6/10	4/10 vs 3/10	1/10 vs 0/10	3/10 vs 2/10	5/10 vs 7/10	1/10 vs 1/10	NR	NR	NR	NR

*NR*, not reported

*Mean ± standard deviation or median (interquartile range)

### Assessment of risk of bias in included studies

The outcomes of risk of bias assessment using Cochrane collaboration’s tool and ROBINS-I tool are presented in Tables 3 and 4, respectively.

**Table 3:** Results of risk of bias assessment of the included randomised controlled trials using Cochrane risk of bias tool

Risk of bias assessment domain	Included studies		
	Yamashita 2021	Takata (2) 2015	Yamamoto 2009
Random sequence generation (selection bias)	Low risk	Low risk	Unclear
Allocation concealment (selection bias)	Low risk	Low risk	Low risk
Blinding of participants and personnel (performance bias)	Low risk	Low risk	Low risk
Blinding of outcome assessment (detection bias)	Low risk	Low risk	Low risk
Incomplete outcome data (attrition bias)	Low risk	Low risk	Low risk
Selective reporting (reporting bias)	Low risk	Low risk	Low risk
Other bias	Low risk	Low risk	Low risk

**Table 4:** Results of risk of bias assessment of the included observational studies using ROBINS-I tool

Risk of bias assessment domain	Included studies	
	Yamashita (2) 2021	Takata 2015
Bias due to confounding	Low risk	Low risk
Bias in selection of participants into the study	Low risk	Low risk
Bias in classification of interventions	Low risk	Low risk
Bias due to deviations from intended intervention	Low risk	Low risk
Bias due to missing data	Low risk	Low risk
Bias in measurement of outcomes	Low risk	Low risk
Bias in selection of the reported result	Low risk	Low risk

### Outcomes (Fig. 2)

#### CRP level postoperative day 3

Analysis of 172 patients from four studies showed that the level of CRP on day 3 post oesophagectomy was significantly lower in the ghrelin group (MD: − 3.64, 95% CI − 5.35 to 1.92, *P* < 0.0001). A low level of between-study heterogeneity was identified (*I*^2^= 0%, *P* = 0.074).

**Fig. 2:**
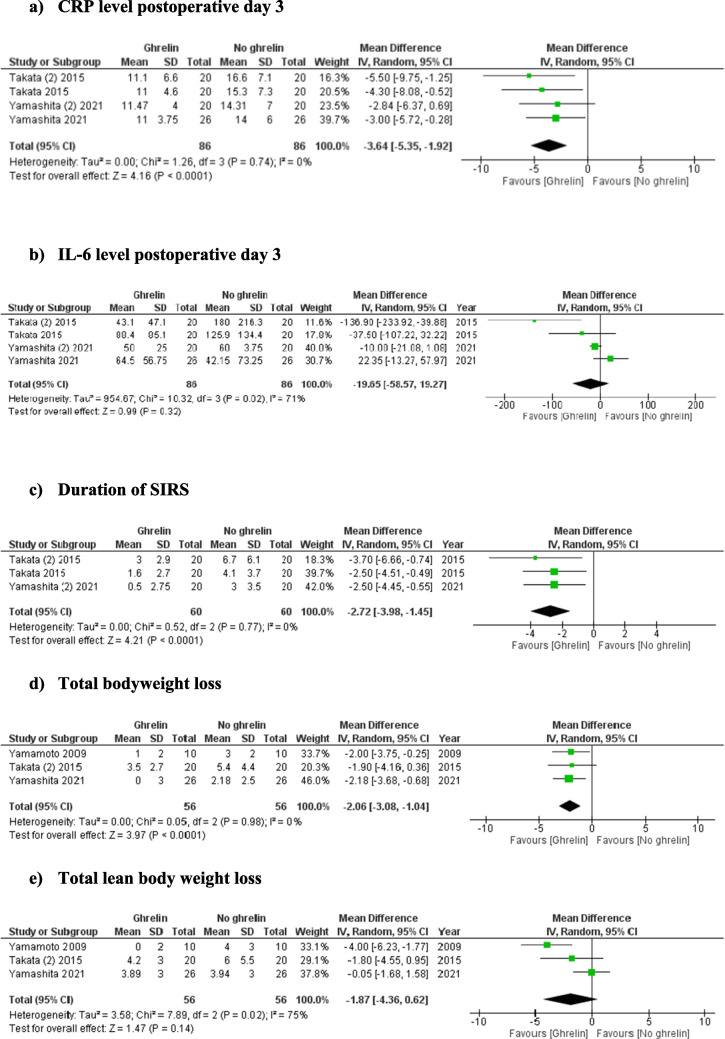

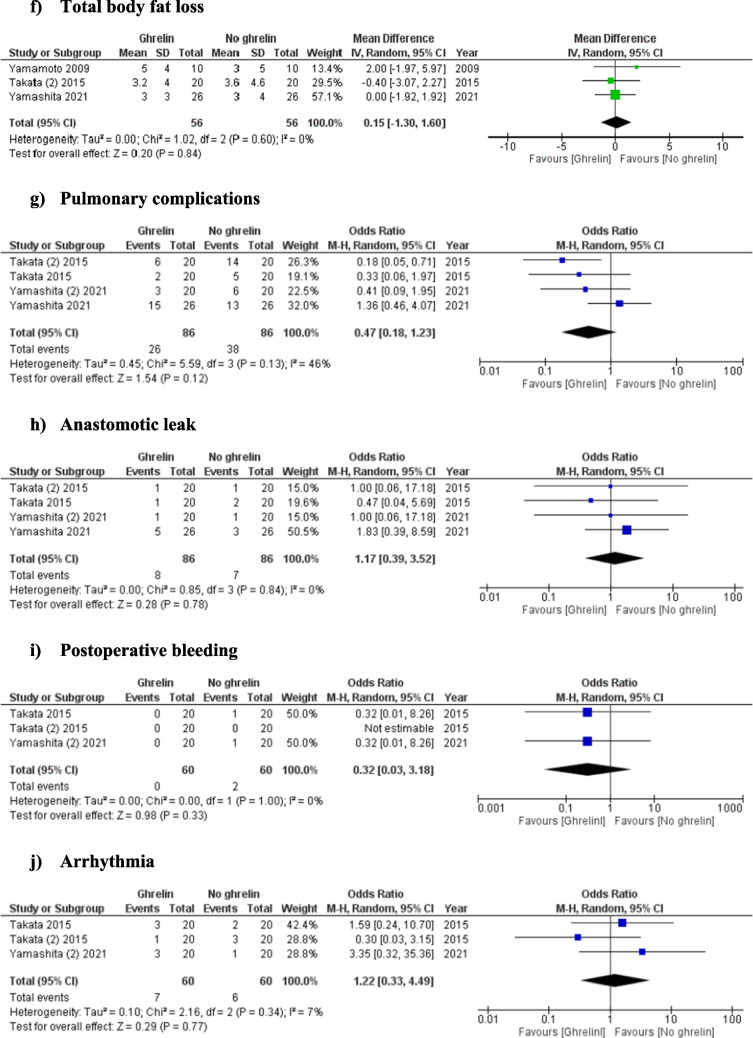
Forest plot for comparison of frequency of adverse outcomes between the ghrelin therapy and no ghrelin therapy groups

#### IL-6 level postoperative day 3

Four studies (172 patients) reported data about IL-6 level on postoperative day 3; meta-analysis showed no significant difference in IL-6 level on postoperative day 3 between the two groups (MD: − 19.65, 95% CI − 58.57 to 19.27, *P* = 0.32). Moderate heterogeneity among the studies existed (*I*^2^ = 71%, *P* = 0.02).

#### Duration of SIRS

Duration of SIRS was reported in three studies (120 patients). The patients who received postoperative ghrelin therapy had a lower duration of SIRS than patients who received no ghrelin or a placebo (MD: − 2.72, 95% CI − 3.98, − 1.45, *P* = 0.0001). Low heterogeneity among the selected studies was identified (*I*^2^ = 0%, *P* = 0.77).

#### Total bodyweight loss

Analysis of 112 patients from three studies showed that total percentage bodyweight loss was lower in the group of patients who received ghrelin therapy postoperatively following oesophagectomy (MD: − 2.06, 95% CI − 3.08 to 1.04, *P* < 0.0001). The level of between-study heterogeneity was low (*I*^2^= 0%, *P* = 0.98).

#### Total lean body weight loss

Three studies reported information regarding the impact of ghrelin administration on lean bodyweight loss. There was no significant difference in the percentage of lean bodyweight lost between the two groups (MD: − 1.87, 95% CI − 4.36 to 0.62, *P* = 0.14). A high heterogeneity among the selected studies was identified (*I*^2^ = 75%, *P* = 0.02).

#### Total body fat loss

Analysis of three studies (112 patients) showed no significant difference in the percentage of body fat loss following oesophagectomy between the patients who received ghrelin therapy and those who did not (MD: 0.15, 95% CI − 1.30 to 1.60, *P* = 0.84). The level of between-study heterogeneity was low (*I*^2^= 0%, *P* = 0.60).

#### Pulmonary complications

Analysis of 171 patients from four studies showed no significant difference in the risk of pulmonary complications between the patients receiving postoperative ghrelin and those who did not receive it. (OR: 0.47, 95% CI 0.18–1.23, *P* = 0.12). The level of between-study heterogeneity was moderate (*I*^2^= 46%, *P* = 0.13).

#### Anastomotic leak

Four studies reported the incidence of anastomotic leak (172 patients), the incidence of which was not different between patients receiving ghrelin therapy and those who did not (OR: 1.17; 95% CI, 0.39–3.52; *P* = 0.78). The heterogeneity among the studies was categorised as low (*I*^2^ = 0%, *P* = 0.84).

#### Wound complications

The incidence of wound complications following oesophagectomy was reported by three studies (120 patients). Wound complications were equally likely to occur in patients receiving ghrelin as those not taking ghrelin therapy (OR: 1.64, 95% CI 0.22–12.45, *P* = 0.63). Heterogeneity among the studies was moderate (*I*^2^ = 45%, *P* = 0.16).

#### Postoperative bleeding

Postoperative bleeding was reported in three studies (120 patients). There was no significant difference in the risk of postoperative bleeding found between the patients who received postoperative ghrelin therapy and patients received no ghrelin or a placebo (OR: 0.32, 95% CI 0.03–3.18, *P* = 0.33). Low heterogeneity among the selected studies was identified (*I*^2^ = 0%, *P* = 1.00).

#### Arrhythmia

Analysis of 120 patients from three studies showed no significant difference in the risk of arrhythmia between the patients receiving postoperative ghrelin and those who did not receive it (OR: 1.22, 95% CI 0.33–4.49, *P* = 0.77). The level of between-study heterogeneity was low (*I*^2^ = 7%, *P* = 0.34).

### Sensitivity analyses

Sensitivity analyses were carried out for CRP level on postoperative day 3, IL-6 level on postoperative day 3, pulmonary complications, and anastomotic leak which had been reported by four studies. When one study was eliminated at a time, the overall conclusion for any of the outcomes was not affected. Repeated analysis of each outcome changing the summary measure from OR to RR and RD did not affect the conclusions for dichotomous outcomes. Finally, separate analyses of randomised controlled trials and studies with low risk of bias confirmed consistency of the findings.

## Discussion

After oesophagectomy in patients with oesophageal cancer, an endogenous decrease in the production of ghrelin can worsen patient morbidity and outcomes such as significant weight loss [19] and systemic inflammation. In this study we conducted a comprehensive systematic review with meta-analysis in order to investigate the role of postoperative ghrelin therapy in patients with oesophageal cancer undergoing an oesophagectomy. Our analysis of five studies reporting 192 patients suggested that the use of postoperative administration of ghrelin may be beneficial as indicated by a shorter duration of SIRS, a lower postoperative level of CRP, and a decrease in the total percentage of bodyweight loss in patients who received postoperative ghrelin therapy. These results remained consistent through sensitivity analyses.

As far as we are aware, this study is the first meta-analysis that has investigated the effect of postoperative ghrelin therapy on the inflammatory response and bodyweight loss in patients with oesophageal cancer undergoing oesophagectomy. Therefore, we cannot compare our findings directly with the findings of studies with similar design. The reduced duration of SIRS and bodyweight loss in the ghrelin therapy group found in the current study and the studies by others is likely due to replacement of ghrelin which is inevitably decreased following oesophagectomy due to decrease in endogenous production of plasma ghrelin [20]. In fact, the concentrations of plasma ghrelin following oesophagectomy are found to decrease by almost 40% of the pre-operative levels [9]. The well-known role of ghrelin is to stimulate hunger [21]; a postoperative drop in this gastric hormone explains the lack of hunger, hence body weight loss after surgery. Ghrelin is also found to inhibit Th1 cells and increase the polarisation of Th2 and regulatory T cells. These actions contribute to the reduced levels of proinflammatory cytokines and increased levels of anti-inflammatory cytokines [22]. All of these could explain the shorter duration of SIRS and lower postoperative CRP level found in the ghrelin group.

Although ghrelin therapy resulted in a shorter duration of SIRS, a lower postoperative level of CRP, and a decrease in the total percentage of bodyweight loss, it did not affect the risk of morbidity outcomes such as pulmonary complications, wound complications, anastomotic leak, or arrhythmia. It can be argued that our findings regarding the morbidity outcomes may be subject to type 2 error due to the relatively small sample size of the included studies. Therefore, it remains unanswered whether shorter duration of SIRS and less bodyweight loss resulted from postoperative ghrelin therapy can translate into improved morbidity outcomes. The lack of evidence on benefits of ghrelin therapy in terms of clinical morbidity outcomes may be a barrier against routine use of ghrelin therapy in patients undergoing oesophagectomy; therefore, there is a need for randomised controlled trials with robust statistical power to investigate the role of postoperative ghrelin therapy on morbidity and mortality outcomes in patients undergoing oesophagectomy.

Weight loss can be considered marker of malnutrition after oesophagectomy and severe weight loss is associated with poor prognosis [23]. It has been shown that the following factors can contribute to weight loss following oesophagectomy: poor eating function, stress response, and gut hormone secretion disorder. [23]. Wang et al. [23] showed that the risk factors for short-term and long-term severe weight losses after oesophagectomy are different. Preoperative sarcopenia, age ≥ 70 years, and vocal cord palsy were considered risk factors for short-term weight loss, while high ASA status, high fat-free body mass, and vocal cord palsy contributed to long-term severe weight loss [23]. Park et al. [24] showed that initial body weight and postoperative vocal cord palsy were risk factors for long-term weight loss after oesophagectomy, while operation-related factors (minimally invasive approach, route of reconstruction, conduit type), postoperative and anastomotic complications, and adjuvant therapy were not significant risk factors [24]. In another study, Schandl et al. [25] identified body mass index at diagnosis, preoperative weight loss, and neoadjuvant therapy as independent predictors of severe weight loss after oesophagectomy [25]. All of the above suggest that weight loss after oesophagectomy is multifactorial and warrants the need for intensive nutritional interventions and monitoring. Ghrelin therapy may address only one of the several risk factors which may result in a smoother postoperative course [26]. On the other hand, it has been shown that continuous ghrelin administration may attenuate skeletal muscle loss during postoperative starvation [27]. This can potentially result in less pulmonary complications, quicker improvement in functional status, and increased likelihood of a full recover which is required for receiving adjuvant therapy [26, 27].

Any interpretation of these results should be tempered by the strengths and limitations present in our study. The points of strengths in the current study include similar baseline characteristics for both groups investigated in the included populations and low between-study heterogeneity for most of the outcomes. The included patients in the ghrelin group and no ghrelin group were comparable in terms of baseline characteristics. This suggests that the results of current study were not influenced by contributing factors such as grade and location of tumour, operative time, or intraoperative blood loss. One of the main limitations of current study was heterogeneity in doses and regimens of ghrelin administration used among the included studies, ranging from 0.5 to 3 μg/kg, either continuously or twice daily, for between 5 and 10 days postoperatively. A limited number of suitable studies available for analysis was another limitation of this study. This not only would subject the findings of the current study to type 2 error but also resulted in inability to comment on the risk of publication bias as we included less than 10 studies. The included studies provided limited information about tumour histology and agents used for neoadjuvant chemotherapy. Finally, the available evidence is limited to studies from a same country conducted by almost the same research group which may affect generalisability of the findings.

## Conclusions

Administration of ghrelin following oesophagectomy may reduce duration of postoperative SIRS and bodyweight loss. Whether shorter duration of SIRS and less bodyweight loss resulted from postoperative ghrelin therapy can translate into improved morbidity or mortality outcomes remains unknown. The available evidence is limited to studies from a same country conducted by almost the same research group which may affect generalisability of the findings. There is a need for randomised controlled trials with robust statistical power to investigate the role of postoperative ghrelin therapy on morbidity and mortality outcomes in patients undergoing oesophagectomy.

## Appendix I

**Table:**

Search strategy^†^	
#1	ghrelin: TI,AB,KW
#2	MeSH descriptor: [ghrelin] explode all trees
#3	#1 OR #2
#4	esophagectom*: TI,AB,KW
#5	oesophagectom*: TI,AB,KW
#6	MeSH descriptor: [esophagectomy] explode all trees
#7	#4 OR #5 OR #6
#8	#3 AND #7

† This search strategy was adopted for following databases: PubMed, MEDLINE, CENTRAL, EMBASE, and CINAHL
